# Exploring the potential of myo-inositol in thyroid disease management: focus on thyroid cancer diagnosis and therapy

**DOI:** 10.3389/fendo.2024.1418956

**Published:** 2024-09-12

**Authors:** S. Adeleh Razavi, Mohadeseh Kalari, Tahereh Haghzad, Fatemeh Haddadi, Shirzad Nasiri, Mehdi Hedayati

**Affiliations:** ^1^ Cellular and Molecular Endocrine Research Center, Research Institute for Endocrine Sciences, Shahid Beheshti University of Medical Sciences, Tehran, Iran; ^2^ Department of Biochemistry, Semnan University of Medical Sciences, Semnan, Iran; ^3^ Department of Biology, Faculty of Sciences, University of Guilan, Rasht, Iran; ^4^ Medicinal Plants and Drugs Research Institute, Shahid Beheshti University, Tehran, Iran; ^5^ Department of Surgery, Shariati Hospital, School of Medicine, Tehran University of Medical Sciences, Tehran, Iran

**Keywords:** thyroid carcinoma, thyroid nodule, myoinositol, cancer diagnosis, biomarker, metabolomics

## Abstract

Thyroid cancer (TC) is a malignancy that is increasing in prevalence on a global scale, necessitating the development of innovative approaches for both diagnosis and treatment. Myo-inositol (MI) plays a crucial role in a wide range of physiological and pathological functions within human cells. To date, studies have investigated the function of MI in thyroid physiology as well as its potential therapeutic benefits for hypothyroidism and autoimmune thyroiditis. However, research in the field of TC is very restricted. Metabolomics studies have highlighted the promising diagnostic capabilities of MI, recognizing it as a metabolic biomarker for identifying thyroid tumors. Furthermore, MI can influence therapeutic characteristics by modulating key cellular pathways involved in TC. This review evaluates the potential application of MI as a naturally occurring compound in the management of thyroid diseases, including hypothyroidism, autoimmune thyroiditis, and especially TC. The limited number of studies conducted in the field of TC emphasizes the critical need for future research to comprehend the multifaceted role of MI in TC. A significant amount of research and clinical trials is necessary to understand the role of MI in the pathology of TC, its diagnostic and therapeutic potential, and to pave the way for personalized medicine strategies in managing this intricate disease.

## 1 Introduction

Inositol is a naturally occurring carbocyclic polyol sugar found in both plant and animal cells, particularly in mammalian cells. Inositol is a key component of essential membrane phospholipids such as phosphatidylinositol (PI), phosphatidylinositol phosphate (PIP), phosphatidylinositol bisphosphate (PIP2), and phosphatidylinositol trisphosphate (PIP3) (1). Additionally, it plays a role in the formation of signaling molecules. Inositol triphosphate (IP3) is a crucial intracellular messenger produced from PIP2 in response to specific hormones or neurotransmitters, which then triggers subsequent signaling pathways (1, 2).

Inositol has different stereoisomers, with myo-inositol (MI) being the most common and studied isomer in the human body. MI is obtained through both endogenous biosynthesis and dietary intake, with high amounts found in various foods. MI plays important roles in cell membranes, brain tissue, kidney medulla, glucose homeostasis, insulin signaling, and neurotransmitter signaling pathways (3, 4). Its involvement in diseases such as anxiety and depression (5), polycystic ovary syndrome (PCOS) (6), diabetes (7), neurological disorders (8), and cancer (9) has been extensively studied. Despite the great importance of MI, it has received less attention in thyroid research compared to other diseases. Although metabolomics studies have identified MI as a biomarker for thyroid lesions, the role of MI in the normal physiology of the thyroid and its effects on the development of thyroid diseases are not yet fully understood. Furthermore, the therapeutic effects of MI in treating thyroid-related diseases, especially thyroid cancer (TC), have not been extensively investigated. The purpose of this review is to collect the published findings of studies that have explored the connection between thyroid diseases and MI. Specifically; this research will discuss the potential physiological and pathological functions of MI, as well as its potential therapeutic applications in the context of thyroid diseases, especially TC.

## 2 Chemistry

### 2.1 Inositols

Inositols are isomers of cyclohexanehexol (cyclohexane-1,2,3,4,5,6-hexol), having the same empirical formula of C_6_H_12_O_6_ (10). Inositols are a unique class of natural metabolites that can be classified as polyols or cyclitols. Their basic structure consists of a cyclohexane with a hydroxyl group bound to each carbon atom of the hexanic ring. Consequently, they are also referred to as 1,2,3,4,5,6-cyclohexanehexols (11, 12). Their structure is very similar to the cyclic form of monosaccharides such as glucose or other hexoses, and for this reason, they are also known as sugar alcohols (13).

**Figure 1:**
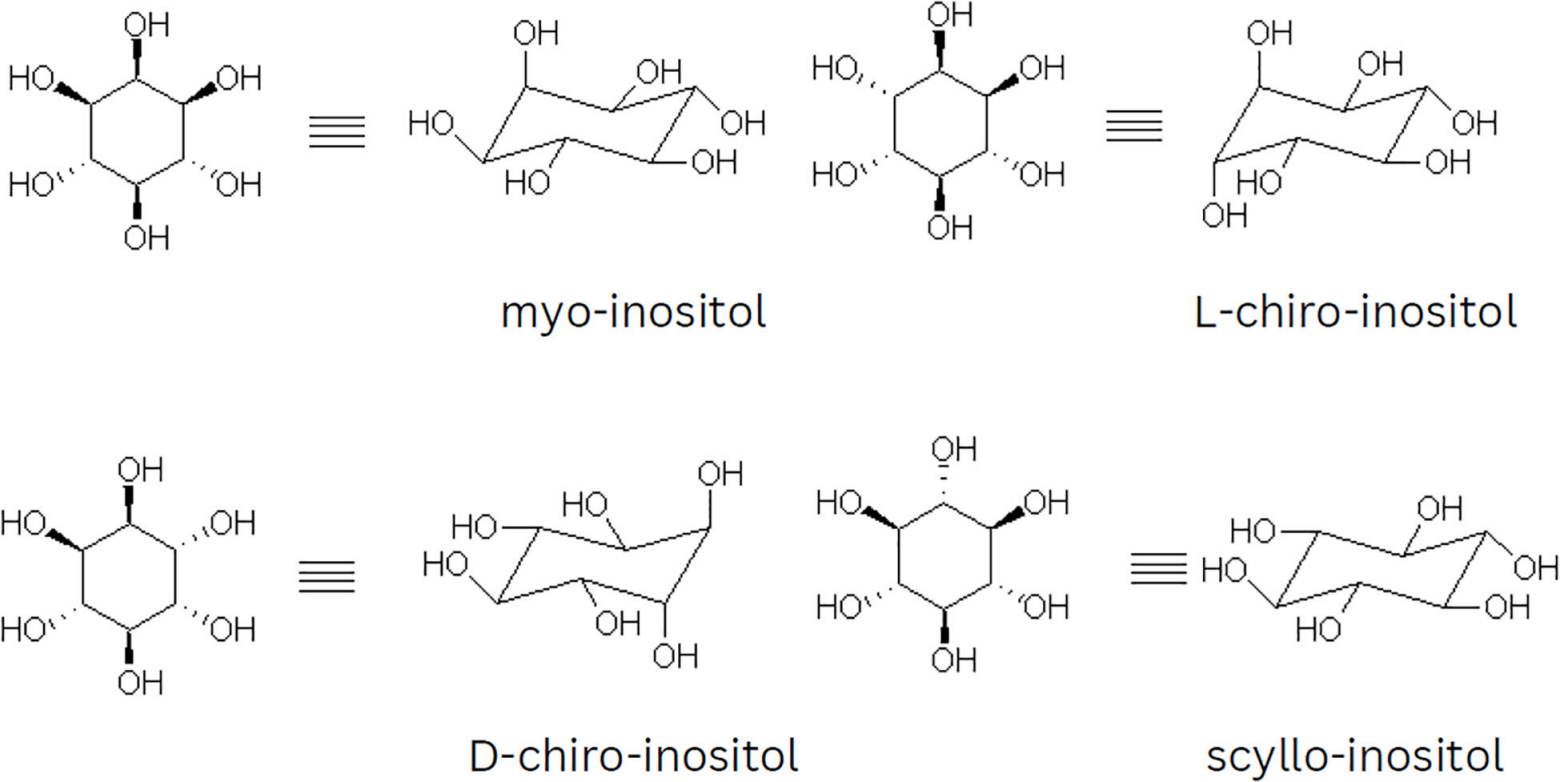
Illustration of the chair conformations and two-dimensional projections of myo, chiro, and scyllo-inositols.

There are nine possible stereoisomers of inositol, each named based on the spatial orientation of its six hydroxyl groups. These include cis-, epi-, allo-, myo-, muco-, neo-, (+)-chiro- (-),-chiro-, and scyllo-inositols (7, 14). Although there are 64 potential stereoisomers with six chiral centers, only nine are possible due to symmetry reasons. Seven of these isomers are optically inactive or meso compounds because they have an internal plane of symmetry. The remaining two isomers, (+)-chiro and (-)-chiro, do not have this symmetry and form an enantiomeric pair. MI is the most common naturally occurring form, but chiro- and scyllo-inositols are also noteworthy. **Figure 1** shows the two-dimensional structures and chair conformations of these isomers.

### 2.2 Myo-inositol

MI is the most abundant and significant compound in the inositol family. Other inositols including stereoisomers and derivatives are the products of metabolic processes on this unique molecule. MI with the IUPAC name of (1R,2R,3r,4S,5S,6s)-cyclohexane-1,2,3,4,5,6-hexol and traditional name of L-inositol has an average molecular weight of 180.1559 g/mol (1). Due to its molecular formula C_6_(H_2_O)_6_, MI can be classified as a carbohydrate (15). In a research study that investigated the solubility of MI in five different solvents (water, methanol, ethanol, isopropanol, and acetone), it was found that the solubility of MI in pure solvents increases as the temperature rises. The highest solubility of MI was observed in water, indicating that water can be used as an effective positive solvent. On the other hand, the other four solvents can be used as anti-solvents due to their low solubility. Since ethanol is considered to be more environmentally friendly than the other three anti-solvents, a binary solvent system of water and ethanol is the preferred choice for crystallization (16).

**Figure 2:**
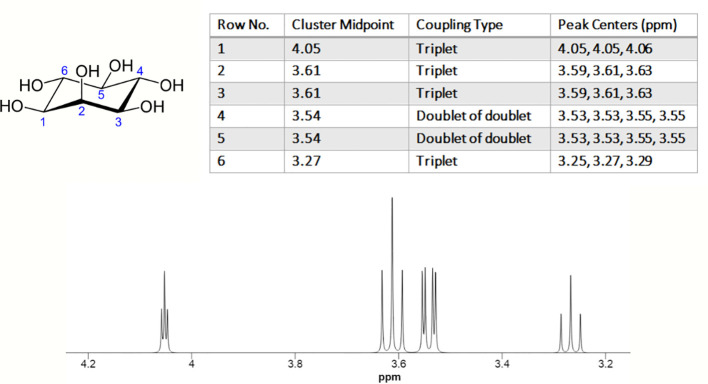
1H NMR spectrum of myo-inositol. The experimental conditions are as follows: solvent: H_2_O; nucleus: 1H; frequency: 600 MHz; sample temperature: 25.0°C, chemical shift reference: DSS. (https://hmdb.ca/).

The nuclear magnetic resonance (NMR) spectrum of MI typically displays 4 peaks, corresponding to the 6 protons present in the molecule. Due to the MI ring’s symmetry around the H2-H5 axis, the number of unique proton resonances detected in MI spectrum is four. The chemical shifts of the peaks are typically observed between 3.2-4.2 ppm (**Figure 2**) (17).

MI can be transformed into several derivatives by epimerization, phosphorylation, or methylation of its hydroxyl groups. However, some of these derivatives cannot be produced in animal cells, and are instead synthesized artificially. In cells, MI exists in various phosphorylated forms, ranging from monophosphorylated forms such as inositol-1-phosphate, inositol-3-phosphate, or inositol-4-phosphate, to the hexaphosphorylated form (IP6) called phytic acid. It should be noted that mono-, di-, and tri-phosphorylated forms are not directly synthesized from the MI phosphorylation in human cells. Instead, these forms can be produced by the dephosphorylation of more phosphorylated forms by specific phosphatases or by phosphoinositide hydrolysis (7).

## 3 Biosynthesis

**Figure 3:**
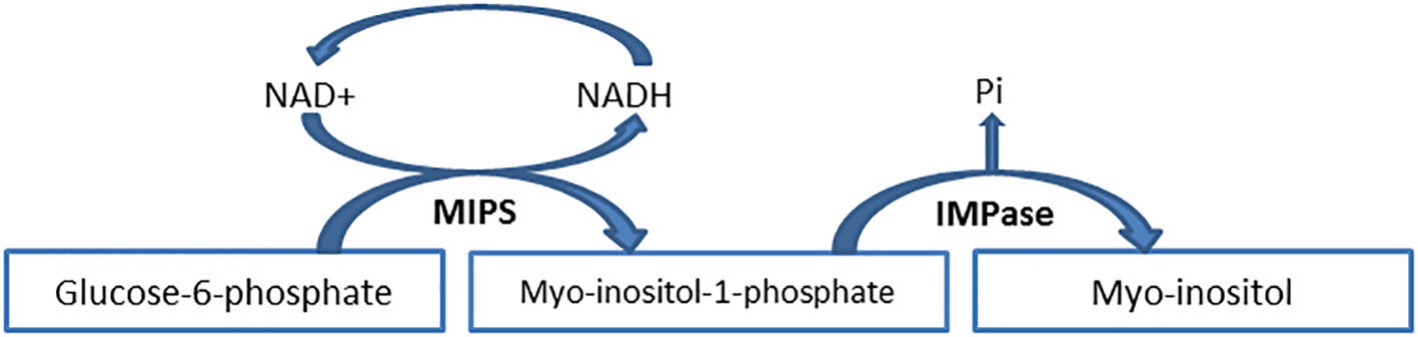
Myo-inositol *de novo* biosynthesis from glucose-6-phosphate. The process of glucose-6-phosphate to myo-inositol-1-phosphate conversion follows a series of consecutive reactions that involve the reduction of NAD+ to NADH. Finally, NADH is oxidized to NAD+ when the process is completed. IMPase inositol monophosphatase, MIPS myo-inositol phosphate synthase, NAD nicotinamide adenine dinucleotide, NADH nicotinamide adenine dinucleotide hydrogen, Pi inorganic phosphate.

MI is synthesized from glucose-6-phosphate (G6P) according to a process requires the assistance of two enzymes: 1) Myo-inositol phosphate synthase (MIPS) or inositol SYNthase A1 (ISYNA1) or Ino1 (in budding yeast), which converts G6P to myo-inositol-1-phosphate (MI1P) (7); 2) Inositol monophosphatase (IMPase or IMPA) which dephosphorylates MI1P to create MI (**Figure 3**). It should be noted that some articles declare that G6P is converted into myo-inositol-3-phosphate (MI3P) due to MIPS activity (18). The enzymes MIPS and IMPase function in unison to allow for the endogenous production of MI in various species.

Many organisms have the ability to biosynthesize MI *de novo*. The utilization of this characteristic is in artificial MI biosynthesis (7, 18). An illustration of this is a study conducted by Yi et al. in 2020. They showed that the biosynthetic pathway of MI was successfully established in *Escherichia coli* by introducing the myo-inositol-1-phosphate synthase-encoding gene (INO1) from *Saccharomyces cerevisiae* S288C. This study has revealed that the quantity of MI generated is directly influenced by the initial concentration of glucose. With an initial glucose concentration of 30 g/L, the optimal level of MI concentration was reached, resulting in a yield of 887 mg/L. However, increasing the initial glucose concentration to 40 g/L resulted in a notable accumulation of acetic acid, leading to a minor decrease in the MI concentration. A final yield of 797 mg/L of MI was obtained from a 1 L shake flask culture, initiated with an initial glucose concentration of 10 g/L (19).

MI is a valuable compound with a wide range of therapeutic benefits. However, the chemical synthesis of MI is not cost-effective. Obtaining MI from natural sources has been shown to have low yields and enhancement is a costly and time-consuming process. Hence, the generation of MI via the biosynthetic route emerges as a highly attractive option. Thus far, MI biosynthesis has been accomplished via enzymatic, microbial, and green synthesis approaches. The utilization of microbial synthesis methodology exhibits remarkable potential in facilitating the extensive production of MI as a result of its exceptional selectivity and economic viability. This section elucidated the outcomes of MI biosynthesis as reported in some recent articles. In an effort to circumvent verbosity, the findings of ten additional investigations have been succinctly summarized in the **Table 1**.

**Table 1:** Approaches and efficiency of myo-inositol (MI) biosynthesis in recent studies.

Biosynthesis Approach	Engineered Strain/ Cell Used	Yield of Biosynthesis	Key Findings	Ref
Metabolic engineering, pathway optimization	*Cupriavidus necator* H16	520.2, 1076.3 and 1054.8 mg/L from glucose, glycerol and CO_2_, respectively	*Cupriavidus necator* H16 was engineered for heterotrophic and autotrophic production of MI	(20)
Metabolic engineering, pathway optimization, metabolic control	*Synechococcus elongates* UTEX 2973	262.6 mg/L	Achievement the CO2-based MI biosynthesis to the same order of magnitude as heterogenous microorganisms with suitable scale-up in the future	(21)
Enzyme engineering, bioprocess optimization	Biomimetic mineralized microcapsules	Inositol titer of up to 210 g/L	Development of a co-immobilized four-enzyme cocktail for large-scale inositol production with increased enzyme lifetime and reduced cost	(22)
Metabolic engineering, dynamic metabolic control, bioprocess optimization	*Pichia pastoris*	30.71 g/L	Dynamic regulation of central metabolism enhanced MI production	(23)
Enzyme engineering, bioprocess optimization	Engineered whole-cell immobilized with colloidal chitin	110.8 g/L inositol with 92.3% conversion	Design of a tri-enzymatic cascade route using novel enzymes for inositol production from glucose Achievement of high titers and conversion from immobilized cells	(24)
Metabolic engineering, pathway optimization, bioprocess optimization	*Escherichia coli*	76 g/L	Glycerol co-utilization enhanced MI production	(25)
Metabolic engineering, pathway optimization, bioprocess optimization	*Escherichia coli*	0.96 mol MI/mol glucose	Production of MI with a high stoichiometric yield and titer	(26)
Metabolic engineering, pathway optimization, bioprocess optimization	*Synechocystis* sp. PCC 6803	12.72 mg/L	Production of MI with a substantial 12-fold increase when compared to the initial MI-producing strain	(27)
Enzyme engineering	*Arthrobacter* sp., *Trypanosoma brucei*, *Escherichia coli*	90%	Novel trienzymatic cascade efficiently synthesized MI	(28)
Enzyme engineering, bioprocess optimization	*Thermobifida fusca YX*	9.98 g/L MI from 10 g/L glucose	Enhanced thermostability and activity of polyphosphate glucokinase mutant through directed evolution Production of glucose 6-phosphate from glucose and polyphosphate without the need for *in vitro* ATP regeneration	(29)
Enzyme engineering, bioprocess optimization	*Thermotoga maritima, Thermococcus kodakarensis, Archaeoglobus fulgidus, T. maritima*	98.9%	Development of an *in vitro* enzymatic pathway using hyperthermophilic enzymes for starch-to-inositol conversion Successful operation on an industrial scale	(30)
Enzyme engineering, bioprocess optimization	*Thermococcus kodakarensis*, *Archaeoglobus fulgidus*, *Thermotoga maritima*, *Escherichia coli*	2.9 g	An *in vitro* enzyme system was developed for the production of MI from starch	(31)
Enzyme engineering, bioprocess optimization	*Escherichia coli*	0.98 mol	A one-pot, multi-enzyme cascade system was developed for efficient synthesis of MI from sucrose	(32)
Dynamic metabolic engineering	*Escherichia coli*	5.5-fold improvement	Successful regulation of metabolic flux in engineered bacteria was achieved using a pathway-independent quorum-sensing circuit	(33)
Dynamic metabolic engineering	*Escherichia coli*	2-fold improvement	Controlled degradation of a key glycolytic enzyme, phosphofructokinase-I was achieved	(34)

## 4 Metabolism

**Figure 4:**
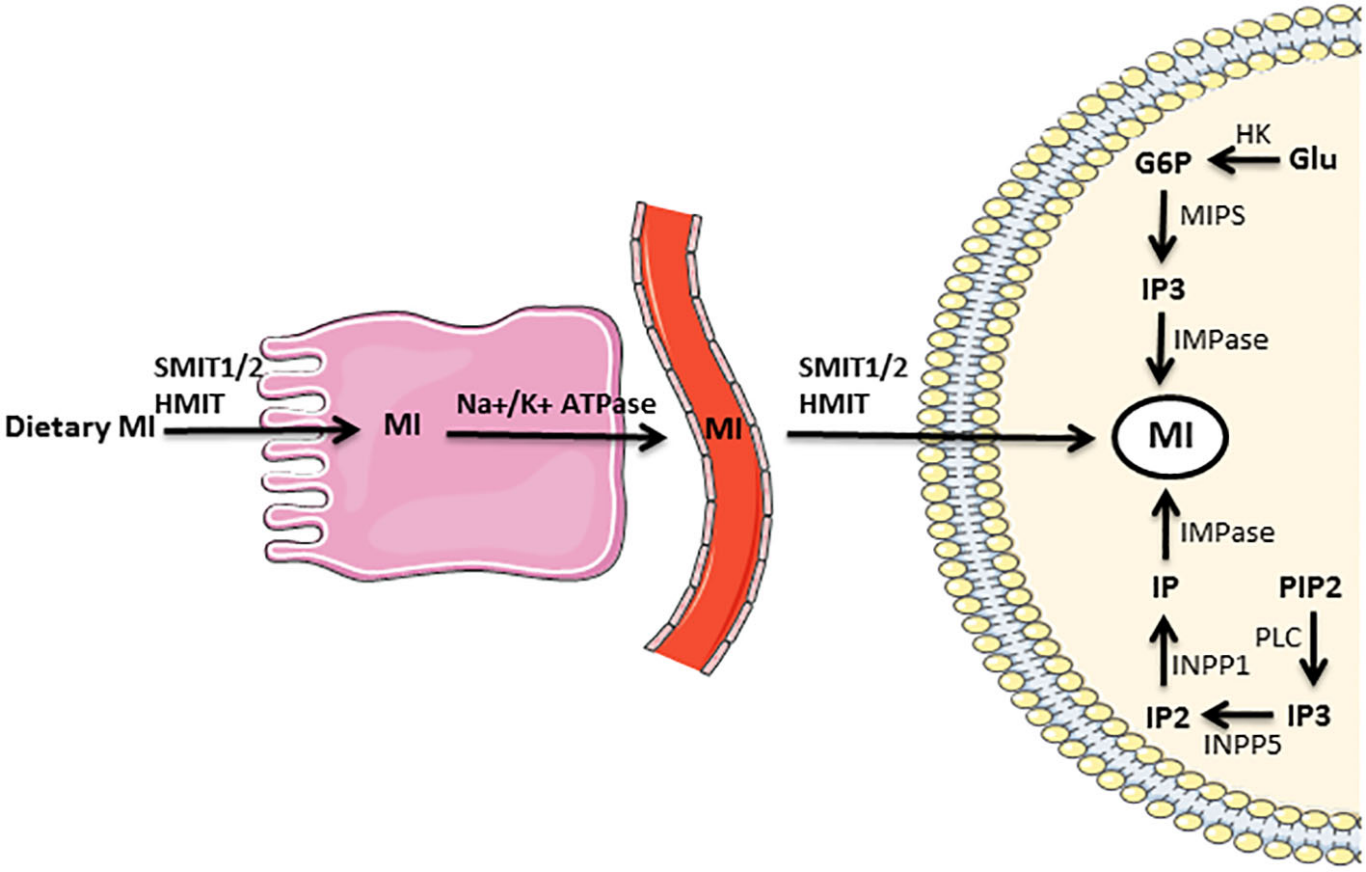
Supply of myo-inositol needed by the cell from three distinct sources: myo-inositol biosynthesis that includes two distinct biochemical reactions, dephosphorylation of IPs, and absorption from the gastrointestinal tract. The full description is in the text. G6P glucose 6-phosphate, Glu glucose, HK hexokinase, HMIT H+-myo-inositol transporter, IMPase inositol monophosphatase, INPP1 inositol polyphosphate-1-phosphatase, INPP5 inositol polyphosphate-5-phosphatase, IP inositol monophosphate, IP2 inositol bisphosphate, IP3 inositol triphosphate, MI myo-inositol, MIPS myo-inositol phosphate synthase, Na+/K+ ATPase sodium–potassium adenosine triphosphatase, PIP2 phosphatidylinositol bisphosphate, PLC phospholipase C, SMIT1/2 sodium-dependent myo-inositol transporters 1 and 2.

The metabolism of MI is a multifaceted biological process that encompasses a variety of enzymatic reactions and pathways, and is precisely modulated by diverse factors, such as hormonal signals, nutrient availability, and environmental stressors. Cells typically obtain MI from three distinct sources (**Figure 4**). First, the synthesis of MI through *de novo* pathways that involves two distinct biochemical reactions (**Figure 3**). The NADH-dependent, cytosolic MIPS isomerizes G6P to MI1P. Then, MI1P undergoes dephosphorylation by IMPase to form free MI. Dephosphorylation of inositol phosphates (IPs) represents another significant pathway for the generation of cellular MI. In brief, each isoform of IP3 is dephosphorylated by a special phosphatase, resulting in the formation of IP2. Subsequently, the action of another phosphatase leads to the conversion of IP2 into MI1P. Finally, MI1P is converted into free MI by IMPase. The third origin of cellular MI is the uptake from the gastrointestinal tract. This process is accomplished through MI transporters that are specialized for this purpose (1, 35). Nearly the entire amount of freely ingested MI is absorbed by the human gastrointestinal tract via an active transport mechanism that involves Na+/K+-ATPase (1). The receptors that uptake MI from the extracellular fluid in the target cell membrane are called sodium-dependent myo-inositol transporters 1 and 2 (SMIT1/2), and H^+^-myo-inositol transporter (HMIT) (36).

The catabolism of MI plays a pivotal role in maintaining inositol homeostasis. This process predominantly occurs in the kidney through the enzymatic activity of myo-inositol oxygenase (MIOX), a nonheme iron enzyme that is accountable for the conversion of MI into D-glucuronic acid. The ensuing stages involve the transformation of D-glucuronic acid into D-xylulose-5-phosphate, which subsequently enters the pentose phosphate pathway. The resulting end products are utilized to produce oxidative energy (4, 37).

The metabolic process of MI exhibits a correlation with the glucose metabolism due to the fact that MI can perform as a mimic of insulin. It means that the utilization and uptake of glucose by cells in the body can be augmented by MI. One potential mechanism by which MI may affect glucose metabolism involves the activation of specific signaling pathways in cells that participate in glucose uptake and metabolism. Furthermore, MI may modulate glucose metabolism by controlling the expression of specific genes that are implicated in glucose uptake and metabolism. Currently, the utilization of MI as a supplement is directed towards dealing with diseases that are associated with insulin resistance, notably PCOS and gestational diabetes.

## 5 Connection with the thyroid gland

A study was conducted to investigate the radioactive MI distribution in adult male rats following intraperitoneal injection revealed MI accumulates in various organs such as the kidney, liver, spleen, pituitary gland, and notably the thyroid gland, within a short span of hours post-injection (38). Studies have established that MI is a fundamental constituent of cellular membranes, with a pivotal function in cell morphogenesis, cytogenesis, lipid synthesis, cellular membrane architecture and cellular proliferation (39, 40). It also serves as a precursor to the production of PIPs, which act as a source for various secondary messengers such as diacylglycerol (DAG), inositol-1,4,5-triphosphate (IP3), and phosphatidylinositol-3,4,5-phosphate (PIP3) (2, 41). Thus, the rapid accumulation of MI in the thyroid can be elucidated by these mentioned characteristics.

**Figure 5:**
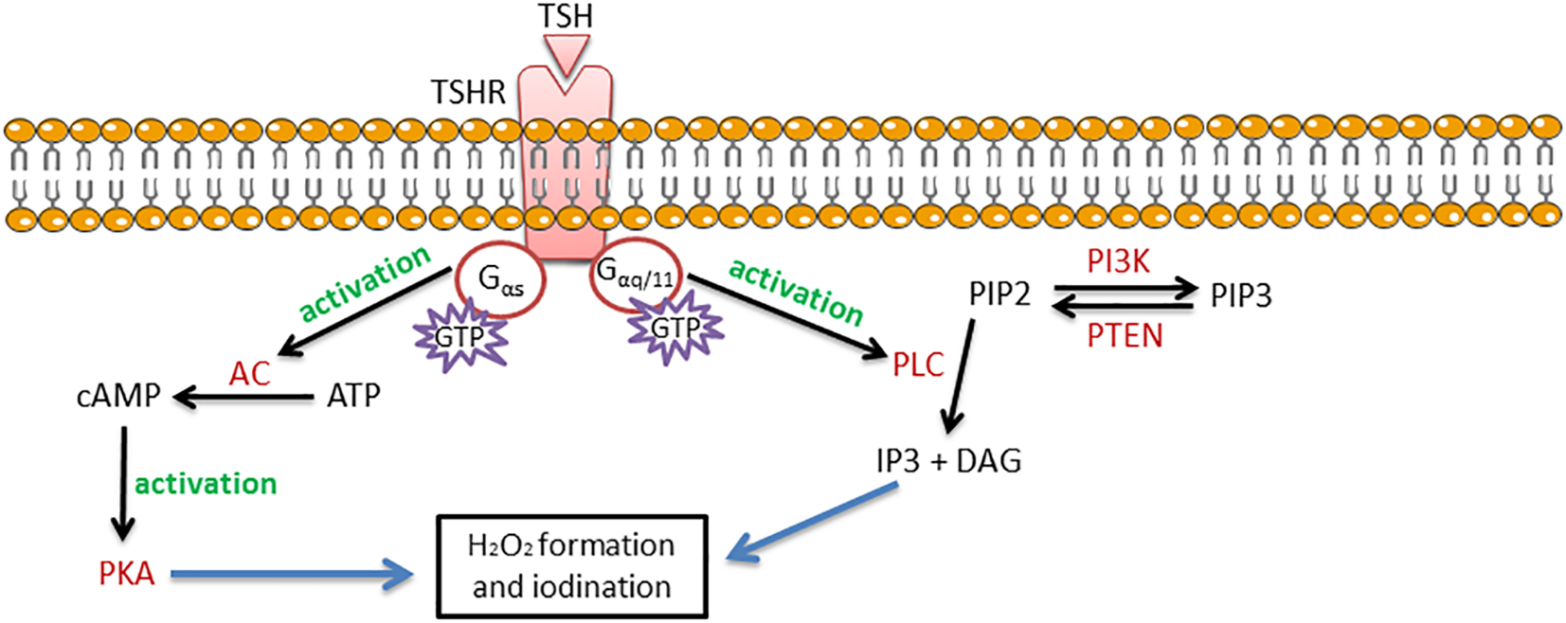
Regulation of thyroid hormone synthesis by two distinct pathways: PIP2 cascade and cAMP cascade. The full description is in the text. AC adenylyl cyclase, ATP adenosine triphosphate, cAMP cyclic adenosine monophosphate, DAG diacylglycerol, GTP guanosine triphosphate, Gαq/11 a subunit of the heterotrimeric G protein, Gαs a subunit of the heterotrimeric G protein, IP3 inositol triphosphate, PI3K phosphoinositide 3-kinase, PIP2 phosphatidylinositol bisphosphate, PIP3 phosphatidylinositol trisphosphate, PKA protein kinase A, PLC phospholipase C, PTEN phosphatase and tensin homolog deleted on chromosome 10, TSH thyroid stimulating hormone, TSHR thyroid stimulating hormone receptor.

The thyroid gland requires the presence of MI for the purpose of synthesizing thyroid hormones (TH). Actually, the synthesis of these hormones is regulated by two distinct pathways, one of which is through phosphatidylinositol-4,5-bisphosphate (PIP2) cascade, while the other is through the cAMP cascade (**Figure 5**). In both of the pathways, at first iodide must be uptaken into thyrocytes, and the binding of thyroid stimulating hormone (TSH) to its receptor (TSHR) must take place. It has been observed that cAMP-mediated signal cascade can be stimulated by relatively low TSH concentration; whereas the inositol-mediated signal cascade requires a 100-fold higher TSH concentration for stimulation (42, 43). When the concentration of TSH is elevated, thyrocytes regulate TH synthesis through TSH/TSHR/phospholipase C (PLC)/IP3. This specific pathway, comparable to the TSH/TSHR/protein kinase A (PKA)/cAMP pathway, regulates the production of H_2_O_2_ and the iodination (42, 43). The production of H_2_O_2_ in a physiological context acts as a limiting factor in the process of iodine organification. When this process is impaired, it can lead to the manifestation of different thyroid health issues.

## 6 Connection with the thyroid diseases

### 6.1 Hypothyroidism

Primary hypothyroidism is a condition that is associated with a reduced or absent physiological functioning of the thyroid gland, which is caused by various intrinsic factors. Central hypothyroidism, on the other hand, is a widely acknowledged hypofunction caused by lesions at the hypothalamus and/or pituitary level. Subclinical hypothyroidism (SCH), also referred to as initial hypothyroidism, is characterized by circulating free thyroxine (fT4) levels that are still within the reference limits, but with elevated serum TSH levels (the value most commonly observed is up to a concentration of 10 mU/L). The malfunctioning of the thyroid gland may result in significant consequences, leading to decreased levels of serum fT4 and increased levels of serum TSH, which is commonly known as frank or overt hypothyroidism (44, 45).

As MI is implicated in the signaling of the TSH hormone and the regulation of iodination leading to TH synthesis, any disturbance to the MI-dependent TSH signaling pathway may result in TSH resistance and hypothyroidism (46). Consequently, the administration of MI may heighten signaling levels and enhance TSH sensitivity. One of the initial study conducted in the discipline encompassed a double-blind randomized controlled trial aimed at assessing the effectiveness of selenium (Se)+MI supplement in SCH patients with autoimmune thyroiditis. In this investigation, a total of 48 SCH females were randomly divided into two distinct groups; one of which was treated with Se (83 µg/day), while the other was treated with Se+MI (83 µg Se+ 600 mg MI/day). The treatment period spanned over a period of six months. In the context of Se+MI consumers group, a significant decrease (31%) in TSH concentration has been detected, while no alterations were discerned in the other group (47).

A study evaluated 642 patients with suspected hypothyroidism and analyzed those who exhibited SCH or displayed borderline TSH levels, attributable to a class I or II thyroid nodules. These participants were administered a daily treatment of a single tablet containing Se and MI for a period of six months, and their outcomes were compared with those of the control group. The findings indicate that in individuals afflicted with SCH who receive Se+MI supplement, there is a decrease in the dimensions, quantity, and elasticity score of thyroid nodules, as well as in TSH levels (48).

A recent study was conducted in Slovakia where 148 SCH patients from 8 different centers were administered a daily dose of 600 mg of MI along with 83 µg of Se for a duration of 6 months. The study participants comprised of female patients of reproductive age with TSH values ranging from 2.5-5 mU/L and anti-thyroid peroxidase antibody (TPOAb)/anti-thyroglobulin antibody (TgAb) positivity, or TSH values ranging from 5-10 mU/L with or without TPOAb/TgAb positivity. This investigation concluded that the incorporation of Se+MI dietary supplementation in the therapy of SCH yields a favorable outcome in terms of restoring TSH levels, alleviating related symptoms, and sustaining thyroid function. In addition, the Se+MI supplementation effectively preserve euthyroid status in individuals with TSH in the reference range still, having a positive attitude towards the TPOAb/TgAb (49).

A parallel outcome was discerned among pregnant females who daily took Se+MI supplementation from the first visit throughout pregnancy. In comparison with pregnant females who abstained from receiving Se+MI, the concentrations of TSH, free triiodothyronine (fT3), and fT4 were maintained in the normal range in treated females leading to preventing SCH (50). As pregnancy can potentially cause elevated TSH levels and result in SCH and complications for both the mother and the fetus, it becomes crucial to decrease the prevalence of SCH during pregnancy. Based on the research conducted by Porcaro et al. (50), it has been found that a combination therapy of Se+MI can effectively prevent hormonal fluctuations related to SCH and maintain the euthyroid state in pregnant women.

There is higher evidence supporting the beneficial effects of MI on individuals suffering from SCH (51, 52). The potential efficacy of MI supplements in hypothyroidism is likely attributed to the existence of various MI derivatives in the TH biosynthesis pathway.

### 6.2 Autoimmune thyroiditis

Autoimmune thyroid disease (AITD) demonstrates a strong correlation with lymphocyte activation, similar to other autoimmune diseases. The regulation of diverse facets of lymphocyte activity is governed by the PI3K, which acts as the pivotal enzyme of the PI3K-AKTpathway. When the ligand binds with the receptor, PI3K catalyzes the production of PIP3, initiating a cascade of events that culminate in a range of cellular responses and immune synapse (53). The PI3K family is segmented into four distinctive categories: Class I, Class II, Class III, and Class IV. These classifications are based on the primary structure, regulation, and lipid substrate specificity observed *in vitro*. Class I PI3Ks are molecular entities comprising of two distinct subunits, namely a regulatory and a catalytic subunit. These subunits exhibit a heterodimeric nature. Additionally, they are categorized into two subsets, namely IA and IB, based on their respective sequence similarities (54). The Class IA PI3K subtype plays a significant role in B cells, and its deficiency results in severe antigenic unresponsiveness. In contrast, both Class IA and IB PI3K are involved in the immune development and function of T cells. Remarkably, T cells deficient in PI3K exhibit unexpected autoimmune characteristics in mice (53, 55). In order to maintain appropriate cellular responses and immune system homeostasis, the PI3K signaling pathway must be accurately regulated, as it is characterized by a complicated network of interdependent interactions.

The identification of AITD was announced by Hakaru Hashimoto, a Japanese physician, in 1912. The disease is marked by the infiltration of lymphocytes in the thyroid gland, which causes the production of anti-thyroid antibodies. AITD encompasses a diverse range of thyroid-related conditions, which span from hypothyroidism, commonly observed in Hashimoto’s thyroiditis (HT), to hyperthyroidism, predominantly observed in Graves’ disease (GD) (56). AITD are categorized as organ-specific autoimmune disorders mediated by T cells due to immune system dysregulation, resulting in an immune attack on the thyroid gland. Lymphocyte infiltrates into the gland are a hallmark feature of AITD (57).

The pathophysiology of AITD has emphasized the crucial role played by chemokines (58). Chemokines are a category of small signaling proteins that perform an indispensable function in the regulation of immune responses and the guidance of immune cell migrations. The impact of chemokines is exerted through the binding to specific G-protein-coupled receptors (GPCRs) situated on target cells. Various chemokine receptors are expressed on distinct types of immune cells. Correspondingly, chemokines are categorized into different families or classes based on structural similarities (59, 60). CXC chemokines constitute a subclass of chemokines that present with a distinctive pair of cysteine amino acids that are separated by an additional amino acid (X). These chemokines possess a tendency to induce the infiltration of neutrophils and are consistently associated with the phenomenon of inflammation (61). C-X-C motif chemokine ligand 10 (CXCL10), also referred to as interferon gamma (IFN-γ)-inducible protein (IP-10), has been associated with the immune-pathogenesis of several autoimmune diseases such as GD via its receptor CXCR3 (chemokine (C-X-C motif) receptor 3) (62). In thyroid tissue, T helper 1 (Th1) lymphocytes have the capacity to induce elevated IFN-γ and tumor necrosis factor alpha (TNF-α) secretion and stimulate CXCL10 production from thyrocytes. As a result, an amplification feedback mechanism is established, which serves to initiate and sustain the autoimmune process (63). Therefore, CXCL10 present in peripheral fluids can be deemed as a distinctive indicator of Th1 type immunity, wherein its levels in circulation are closely associated with the severity and extent of thyroid auto-inflammatory state (64).

Research has demonstrated that cytokines have the capacity to regulate MI within thyroid cells. The investigation conducted by Kung et al. in 1995 reveals that IFN-γ possesses the ability to elevate the concentration of calcium and IPs within the thyroid cells (65). Although the precise impact of MI on cytokines modulation remains to be fully elucidated, clinical investigations have provided evidence suggesting that MI in combination with Se, can confer therapeutic benefits for AITD. A research carried out by Nordio et al. involved 168 patients who were diagnosed with HT indicated that the administration of Se+MI could potentially lead to a decrease in TSH, TPOAb and TgAb levels (66). The aforementioned research team conducted a further investigation concerning 86 HT individuals with SCH who displayed a TSH value ranging between 3 and 6 mIU/L, and were subjected to an increase in serum TPOAb and/or TgAb, and normal fT4 and fT3 levels. The team administered a Se+MI compound to these patients for a period of six months. The findings of this study demonstrated a marked reduction in the TSH, TPOAb, and TgAb levels, and a notable increase in the fT3 and fT4 levels, consequent to the administration of Se+MI (67). A further investigation conducted a clinical trial on 21 Caucasian individuals who were diagnosed with euthyroid chronic autoimmune thyroiditis. The participants received MI plus Se tablets (600 mg/83 µg), twice daily, for a duration of six months. The findings revealed a reduction in the level of TSH, anti-thyroid autoantibodies and CXCL10 in the subjects who were administered the tablets (68). It appears that MI possesses a protective influence on thyroid cells, as evidenced by the research conducted by Ferrari et al. (69). It was discovered that MI reduces the secretion of CXCL10 chemokine induced by IFN-γ and TNF-α in thyroid cells, regardless of the existence or nonexistence of H_2_O_2_ (69).

More recently, a study was conducted to assess the potential of MI in amplifying the protective effects of Se against the advancement of HT to hypothyroidism. The HT patients, who were either euthyroid or SCH, were divided into three groups: untreated, treated with 83 μg/day selenomethionine and treated with 83 μg/day selenomethionine plus 600 mg/day MI. The findings of this study indicate a significant decrease in TSH levels by 31% in the group that received Se and by 38% in the group that received selenomethionine+MI, as compared to the untreated group. In the selenomethionine+MI group, the decrease in TSH was detected at an earlier time point in comparison to the group treated only with selenomethionine. The authors of this study arrived at the conclusion that the administration of Se proves to be efficacious in individuals afflicted with HT, and its impact could potentially be amplified when accompanied by MI (70). In the investigation carried out by Payer et al., there was an improvement in the autoimmune thyroid (AIT) changes after a duration of 3 months through the administration of Se+MI. This study established the AIT index as the sonographic features of the thyroid, where an AIT index with a score of 1 indicated an alteration in thyroid ultrasound, while an AIT index with a score of 0 indicated no alteration in thyroid ultrasound (49).

A modulatory effect of the Se+MI combination on CXCL10 shows that these two compounds can potentially regulate the Th1 immune response. This finding advocates for further investigations into autoimmune disorders that are characterized by a Th1 immune response.

### 6.3 Thyroid cancer

In the year 2020, the International Agency for Research on Cancer (IARC) published a report stating that the age-standardized incidence rates of TC were 10.1 per 100,000 women and 3.1 per 100,000 men. Additionally, the age-standardized mortality rates were reported to be 0.5 per 100,000 women and 0.3 per 100,000 men (71). The traditional categorization of TC involves the five distinct groups: papillary TC (PTC), follicular TC (FTC), Hurthle cell carcinoma (HTC), anaplastic TC (ATC), and medullary TC (MTC) (72). However, the recent update of the WHO classification of endocrine and neuroendocrine tumors has introduced a new classification for thyroid tumors. This new classification provides a more distinct insight into the status of the malignant tumors and encompasses i) PTC, ii) invasive encapsulated follicular variant PTC (IEFV-PTC), iii) FTC, iv) oncocytic carcinoma of the thyroid (OCA), v) differentiated high-grade TC (DHGTC), vi) poorly differentiated TC (PDTC), and vii) ATC. In the new classification, the MTC originating from the thyroid C cells maintains its unique category (73, 74).

PTC and FTC represent the predominant subtypes of TC that arise from follicular cells (75, 76). Factors that increase the risk of developing PTC or FTC include exposure to ionizing radiation, particularly in younger patients, familial TC, or TC-related disorders such as Cowden syndrome (77–79). Moreover, the incidence of PTC and FTC may be impacted by iodine consumption, TSH levels, AITD, gender, estrogen levels, obesity, lifestyle, and environmental toxins (80). It is notable that the majority of PTC and FTC remain asymptomatic during the initial stages of the illness. However, as the malignant mass grows, a variety of symptoms such as hoarseness, dysphagia, lymphadenopathy, and pain in the neck and throat may appear (81). Due to their moderate differentiation, PDTCs exhibit a broad range of biological aggressiveness (82). Burman et al. introduced the concept of PDTC in 1996, describing it as carcinomas of follicular thyroid epithelium that retain enough differentiation to produce scattered small follicular structures and some thyroglobulin, but generally lack the typical morphologic characteristics of papillary and follicular carcinoma (83). ATC, despite its low prevalence accounting for 1% of all thyroid malignancies, is still considered one of the most devastating diseases worldwide due to its high fatality rate and poor prognosis (100% fatality rate) (75, 84). Fortunately, in contrast to tumors that exhibit differentiation, the incidence of ATC has exhibited a gradual decline over the course of recent decades in prosperous nations (75). MTC is a challenging condition of TCs to manage, arises from the parafollicular cells and constitutes 4% of all TCs (84, 85). Although the majority of MTC cases are sporadic, some cases manifest a hereditary trait known as multiple endocrine neoplasia type 2 (MEN 2). The pathophysiological characteristics of MTC are markedly harsher than those of PTC and FTC, thereby leading to a 10-year survival rate of around 50% (86).

The molecular pathology of different types of TCs is distinguishable. Several PTCs are characterized by the presence of mutations and gene rearrangements that facilitate the activation of the mitogen-activated protein kinases (MAPKs) pathway, ultimately leading to cell division. The activation of the MAPK pathway is created by genetic modifications including rearrangement of RET and NTRK1 tyrosine kinases, or active mutations of BRAF or RAS. It is noteworthy that every PTC harbors exclusively one of these genetic alterations (87–90). In the case of FTC, PAX8 and PPARγ rearrangement, as well as RAS active mutations, have a high frequency. Furthermore, the inhibition of PTEN phosphatase leading to the activation of the PI3K pathway has been recognized as an additional contributing factor to the development of FTC (91–94). The presence of TP53 mutation has been widely noted in the majority of PDTC and ATC. Furthermore, a noteworthy contribution to the pathogenesis of ATC has been attributed to the CTNNB1 mutation, which has been reported to occur in up to 65% of ATC cases. It is crucial to note that several ATC cases may be derived from PTC (89, 95, 96). An accurate comprehension of the molecular pathology underlying TC can clarify their heterogeneous clinical manifestations, earlier diagnosis, favorable prognosis, and the identification of efficacious therapeutic modalities.

#### 6.3.1 Diagnosis of thyroid cancer

In recent years, the exploration of metabolomics has been carried out as one of the methodologies to discern novel biomarkers for the purpose of diagnosing TC (97). Metabolomics is a discipline that investigates substrates, intermediates, and end-products of metabolic pathways, which offers a formidable approach as metabolites serve as direct indicators of the biochemical activity and condition of cells and tissues, thereby providing the best representation of the molecular phenotype. This approach is highly valuable given that metabolic pathways are influenced by a range of factors, including diseases. Until the present time, numerous inquiries into the TC metabolomics have been conducted, resulting in the identification of various metabolites for the aim of TC diagnosis. One of the metabolites that can be observed within the identified metabolic profiles is the highly prized compound of MI.

The studies conducted by Miccoli et al. (98) and Torregrossa et al. (99) were two of the initial metabolomics studies to report MI depletion in TC tissues. The findings from these investigations demonstrated a notable decrease in the level of MI in malignant thyroid tissue when compared to benign tissue. A different investigation carried out with NMR-based metabolomics revealed that the MI level is lower in malignant thyroid tissue when compared to all three types of normal tissue, follicular adenoma tissue, and non-neoplastic nodule tissue (100). Subsequent research demonstrated variations in MI levels in different samples of patients with TC. We observed in our recent study that the plasma level of MI in PTC is lower than in healthy subjects (101). Several metabolomics-based investigations, as presented in **Table 2**, have identified MI as a crucial metabolite in individuals diagnosed with TC. Based on the findings of these studies, it appears feasible to conclude that MI could potentially be employed as a diagnostic biomarker for TC. However, what are the possible underlying mechanisms that account for the reduced levels of MI in TC cells?

**Table 2:** Metabolomics studies introduced myo-inositol as a biomarker in thyroid cancer.

No. of participants (female/male)	Sample type	No. and cyto/histopathology of specimens	Approach	Key Findings	Ref
72 (47/25)	Intact tissue & FNAB	28 PTC 40 Indeterminate 4 Benign 28 Normal 12 FNAB	NMR	Downregulated in malignant thyroid tissue than benign thyroid tissue	(98)
72 (47/25)	Intact tissue	27 PTC 1 ATC 10 FTC 30 FA 4 NG 28 Normal	NMR	Downregulated in malignant thyroid tissue than benign thyroid tissue	(99)
31 (4/27)	Tissue extract	16 Non-neoplastic nodule 14 FA 15 Thyroid carcinoma 19 Normal	NMR	Downregulated in malignant thyroid tissue than normal tissue Downregulated in malignant thyroid tissue than FA Downregulated in malignant thyroid tissue than non-neoplastic nodule	(100)
35 (---)	FFPE tissue	7 FTC 8 PTC 6 MTC 6 ATC 3 FA 5 Non-tumoral tissue	GC-MS	Upregulated in malignant thyroid tissue than FA	(102)
77 (44/33)	Serum	37 PTC with distant metastasis 40 PTC with ablation	GC-MS	Downregulated in distant metastasis group than ablation group	(103)
96 (72/24)	FNAB	46 Benign 52 Malignant	NMR	Downregulated in malignant thyroid FNAB than benign FNAB	(104)
334 (---)	Serum Plasma Tissue	141 PTC 93 Benign 100 Healthy	LC-MS	Downregulated in thyroid lesions (PTC and Benign) than healthy	(105)
14 (12/2)	Tissue extract	11 Thyroid carcinoma 5 Benign 5 Normal	NMR	Downregulated in malignant thyroid tissue than benign or normal thyroid tissue	(106)
141 (113/28)	Intact frozen tissue	38 PTC 32 Benign 112 Non-tumoral tissue	NMR	Downregulated in malignant thyroid tissue than benign thyroid tissue	(107)
55 (44/11)	Plasma	20 PTC 16 MNG 19 Healthy	NMR	Downregulated in PTC than healthy plasma	(101)

ATC anaplastic thyroid carcinoma, FA follicular adenoma, FFPE formalin-fixed paraffin-embedded, FNAB fine needle aspiration biopsy, FTC follicular thyroid carcinoma, GC-MS gas chromatography mass spectrometry, LC-MS liquid chromatography mass spectrometry, LN lymph node, MNG multinodular goiter, MTC medullary thyroid carcinoma, NG nodular goiter, NMR nuclear magnetic resonance, PTC papillary thyroid carcinoma.

One of the crucial functions of free MI is its role as an osmolyte, a function that has been conserved throughout evolution (108). In mammals, the presence of inositol is vital for maintaining the osmolarity in organs that are situated within a hyperosmolar environment (109). Given the reduction in MI concentration within TC cells, it is plausible to suggest that the osmolarity equilibrium in these cells differs from that of benign or normal cells.

Cancer cells typically undergo an elevated level of lipid synthesis. MI is a compound possessing the capability to regulate the metabolic process of lipids in cancer cells. Specifically, it serves as a precursor for the synthesis of PI and its derivatives, which are crucial elements of cell membranes. As a result, cancer cells rely on both MI and these derivatives for the facilitation of signaling pathways (3). On the other hand, cancer cells often demonstrate an increased requirement for lipid production to support their rapid growth and proliferation. MI has the potential to influence the production of essential lipids like phospholipids that are vital for the growth and survival of cancer cells (1, 110).

MI plays a considerable role in the PI3K-AKT-mTOR signaling pathway, which is often dysregulated in cancer cells. This pathway regulates a number of important cellular processes such as cell growth and survival. The decrease in MI levels observed in TC may be linked to disruptions in the PI3K-AKT-mTOR pathway (111). Further research on the underlying mechanisms of MI depletion in TC cells could provide valuable insights for improving TC diagnosis.

#### 6.3.2 Treatment of thyroid cancer

One area of cancer research that is currently being explored is the investigation of the therapeutic effects of MI in the treatment of different types of cancer. IP6, an important member of the inositol family, has been identified as exhibiting anticancer properties in various experimental models. In addition to diminishing cell proliferation, IP6 promotes the differentiation of malignant cells, often leading to a restoration of the normal phenotype. The most reliable and optimal anticancer outcomes have been achieved through the concurrent administration of IP6 and inositol. This combination has been shown to augment the anticancer efficacy of traditional chemotherapy, manage cancer metastasis, and enhance the overall quality of life, as evidenced in a preliminary clinical study.

Thus far, numerous roles have been proposed for the efficacy of MI in the treatment and management of cancer, which can be condensed into several categories: 1) Molecular investigations have demonstrated that MI and hexophosphate (MIP6) possess the ability to manifest anti-cancer characteristics through the direct or indirect disruption of PI3K-AKT-mTOR, MAPK, and Wnt/*β*-catenin pathways and modulation of insulin activity involved in cellular signals transduction, which play a crucial role in the survival, proliferation, and metastasis of cancer cells (3). 2) MI has the ability to enhance the efficacy of radiation therapy on cancerous cells. Studies demonstrate that the mediation of tumor oxygenation and vascular protection by myo-inositol trispyrophosphate initiates both immediate and delayed mechanisms to heighten the potency of ionizing radiation, enabling successful radiation therapy (112). 3) Studies have indicated that MI could potentially possess chemopreventive properties and serve as a preventive measure against the progression of cancer in high-risk individuals or populations (113, 114). 4) There is some indication that the supplementation of MI may offer benefits in mitigating specific treatment-related side effects in cancer patients (115, 116).

Among these specific mechanisms hypothesized for the therapeutic efficacy of MI, the role of MI in regulating the release of calcium ions from the thyrocytes endoplasmic reticulum can be worth considering for TC treatment. This process is essential for activating the dual oxidases 1 and 2 (DUOX 1 and 2) system, leading to the production of H_2_O_2_ and iodination. MI potentiates the stimulating effects of TSH while inhibiting the PI3K-AKT-mTOR pathway (117). On the other hand, given the inherent effects of MI on the cytoskeleton, it is conceivable that MI may enhance the stability of cellular architecture and inhibit the motility and invasion of malignant cells (48). These intricate molecular events have the capacity to promote cellular differentiation and prevent the development of cancer. However, the existing gap in knowledge within this domain is extensive and further investigations are warranted.

While the potential capabilities of MI, there is a lack of clinical research supporting the efficacy of MI in the treatment of TC. However, a compelling study explored the potential influence of oral Se+MI on the size of benign thyroid nodules (48). In this research, individuals with SCH or borderline TSH level linked to low-risk or intermediate-risk nodules were chosen. The group serving as the control, which consisted of 16 individuals, did not undergo any form of therapy. On the other hand, the treatment group, which comprised of 18 individuals, was provided with a daily dosage of one tablet containing Se+MI (83μg/600mg) for a duration of six months. After the completion of the treatment period, the size, number, and elasticity of the nodules, along with the TSH levels, were assessed. The study’s results indicated that patients with SCH who received treatment with Se+MI experienced a decrease in the size, number, and elasticity score of thyroid nodules, as well as a reduction in TSH levels (48).

It is possible that the increasing of MI levels in thyroid cells results in elevated TSH sensitivity and consequently a decrease in its concentration in the serum. Some studies have indicated that elevated levels of TSH may be linked to an increased risk of TC (118–120). Given the findings of several studies demonstrating the efficacy of MI in reducing TSH, it is plausible that this supplementary treatment could potentially serve as a preventative action against TC in individuals afflicted with hypothyroidism and autoimmune thyroiditis.

The confirmation of the positive impact of MI on the thyroid of mice that were treated with cadmium (Cd), a carcinogen, further validates the beneficial nature of MI. Benvenga and colleagues have demonstrated that Se+MI effectively protect the thyroid of mouse from hyperplasia and hypertrophy of C-cells induced by Cd (121). Another study suggested that the use of a combination of MI plus Se could protect the thyroid of mice exposed to Cd (122). Despite the limited amount of research conducted, it appears that MI has the potential to act as an anti-cancer agent in models of TC.

The existing research is not extensive enough to reach a definitive conclusion. It is imperative that scientists come together to expand our knowledge on the impact of MI on TC treatment. It is through these endeavors that we are able to enhance our understanding of the effectiveness of MI in TC treatment.

## 7 Conclusion

MI is a crucial natural compound that contributes to the physiological function of the thyroid gland. Extensive research has been conducted to study its therapeutic impact on SCH and AITD. Metabolomics investigations have documented a reduction in the level of MI in cancerous tumors of the thyroid and identified MI as a metabolic biomarker. Although the number of studies on TC is quite limited, it can be asserted that MI possesses the potential to serve as a therapeutic agent for TC without any significant side effects. Nevertheless, further extensive investigations are necessary to ascertain the diagnostic and therapeutic significance of MI in relation to benign thyroid nodules and TC.
